# Experimental light at night explains differences in activity onset between urban and forest great tits

**DOI:** 10.1098/rsbl.2023.0194

**Published:** 2023-09-06

**Authors:** Ciara L. O. McGlade, Pablo Capilla-Lasheras, Robyn J. Womack, Barbara Helm, Davide M. Dominoni

**Affiliations:** ^1^ IGFS, Queen's University Belfast, Belfast BT9 5DL, UK; ^2^ School of Biodiversity, One Health and Veterinary Medicine, University of Glasgow, Glasgow G61 1QH, UK; ^3^ Swiss Ornithological Institute, 6204 Sempach, Switzerland

**Keywords:** circadian rhythms, ALAN, light pollution, *Parus major*, urbanization, incubation

## Abstract

Artificial light at night (ALAN) is rapidly increasing and so is scientific interest in its ecological and evolutionary consequences. In wild species, ALAN can modify and disrupt biological rhythms. However, experimental proof of such effects of ALAN in the wild is still scarce. Here, we compared diel rhythms of incubation behaviour, inferred from temperature sensors, of female great tits (*Parus major*) breeding in urban and forest sites. In parallel, we simulated ALAN by mounting LED lights (1.8 lx) inside forest nest-boxes, to determine the potentially causal role of ALAN affecting diel patterns of incubation. Urban females had an earlier onset of activity compared to forest females. Experimentally ALAN-exposed forest females were similar to urban females in their advanced onset of activity, compared to unexposed forest birds. However, forest females exposed to experimental ALAN, but not urban females, were more restless at night than forest control females. Our findings demonstrate that ALAN can explain the early activity timing in incubating urban great tits, but its effects on sleep disturbance in the forest are not reflected in urban females. Consequently, future research needs to address potential effects of ALAN-induced timing on individual health, fitness and population dynamics, in particular in populations that were not previously affected by light pollution.

## 1. Introduction

Many biological processes are regulated by natural cycles of light and darkness in interaction with biological clocks [1]. It is increasingly recognized that such processes can be affected by artificial light at night (ALAN), a source of anthropogenic pollution that has been rapidly expanding in extent and radiance over the last 150 years [2]. Recent studies have shown that the impacts of ALAN on wild species is profound, with broad effect on physiology and cascading effects on populations and biodiversity [1,3].

Across the kingdom of life, one of the most reported effects of ALAN is the change in the timing of biological rhythms [4]. In birds, a taxon whose biological rhythms are highly studied, previous correlative work has shown that ALAN exposure can advance the morning onset of activity and disrupt sleep [3]. However, correlative field studies cannot separate the effect of ALAN from that of other environmental variables. Potentially confounding effects are particularly relevant along urban gradients, where ALAN levels strongly covary with other features (e.g. noise [5] or ambient temperature [6]). Experimental evidence from captive studies on birds supports correlative field data [7,8], and further suggests that advanced morning activity in response to ALAN can be accompanied by modifications in physiological rhythms [7,9]. By contrast, experimental evidence from the wild is still scarce and contradictory. For example, a few studies showed that ALAN can induce early onset of locomotor activity and dawn song [10–13]. However, no effect of ALAN on timing of dawn song in several bird species was found in one Dutch study where experimental light pollution was applied [14].

Here, we experimentally introduced ALAN in nests of incubating great tit (*Parus major*) females, to determine whether ALAN on its own may explain differences in diel incubation timing between urban and forest bird populations. We studied females during incubation because this is an important life-history stage for fitness [15]. A recent study on great tits showed variation in incubation behaviour between urban and forest populations: despite urban females spending more time incubating than forest females, their eggs experienced the same average, albeit more variable, temperature than the eggs in forest clutches [16]. In tits, females alone incubate the clutch, an activity that they need to trade off against foraging outside the nest [17,18]. Thus, females must balance the need to keep the eggs within the suitable range of temperatures for embryonic development, while also obtaining enough food [19,20]. Females may thus display individualized incubation patterns, and in our study system early rising great tits benefit from higher reproductive success [21]. Males might also influence female incubation pattern and timing as they provide food to the females during incubation [17].

In the urban environment, several factors may interfere with incubation. ALAN may induce earlier termination of night rest, during which diurnal birds usually provide steady heat, or increased restlessness during the night [22]. Reduced food availability in cities [23,24] may also affect the ability of the female to balance her own energetic needs with the thermal requirements of the embryos, leading to reduced clutch attendance. Noise might additionally interrupt nocturnal sleep, leading to increased restlessness [5].

In this study, we compared morning activity onset, evening activity end and nocturnal restlessness in three groups: females in unmanipulated forest sites served as controls under natural light conditions (hereafter ‘forest controls’); a second group of forest females were experimentally exposed to ALAN to test for effects solely due to light pollution (hereafter ‘forest ALAN’); the third group were females that experienced the full urban environment, without experimental manipulation (hereafter ‘urban’). We predict that: (1) activity onset will be earlier in urban and forest ALAN females compared to forest controls, (2) activity offset may be delayed, advanced or unchanged in urban and forest ALAN birds compared to forest controls, and (3) variance in egg temperature during the night-time will be higher in urban and forest ALAN birds compared to forest controls.

## 2. Methods

### (a) Study area

The study took place in the spring of 2018 in three forest sites and two urban sites in Scotland where approximately 500 nest-boxes were monitored for research. The forest sites were: (i) the Scottish Centre for Ecology and the Natural Environment (SCENE) (56° 7′ N, 4° 36′ W), (ii) the Sallochy campsite (56° 7′ N, 4° 36′ W) and (iii) Cashel farm (56° 6′ N, 4° 34′ W). The urban sites were located in Glasgow: (i) Kelvingrove Park (55° 52′ N, 4° 17′ W) and (ii) the Garscube Sports Complex (55° 54′ N, 4° 18′ W). For details on light, noise and vegetation at the sites, see electronic supplementary material, text and table S2).

### (b) Experimental protocol

We used 12 clutches for each of the forest controls, forest ALAN and urban groups (electronic supplementary material, table S1). Forest controls and urban clutches are also a subset of earlier research [21]. Clutches in the forest were assigned alternatingly to the forest control or forest ALAN groups. In the forest ALAN clutches, on the day that the 4th egg was laid, the nest-box containing the whole clutch was replaced by an experimental nest-box into which the clutch (including the nest) was transferred. This nest-box was identical except that a small (3 mm diameter) LED bulb was present on the inside ceiling of the box. The LED emitted a cool white light with an intensity equal to 1.8 lx in all nest-boxes, and was on for the entire 24 h. We disturbed the nest-boxes of the forest control and urban clutches on the equivalent day, although these nests were not moved to a new nest-box. All boxes were monitored at least weekly.

### (c) Determining incubation characteristics

We determined nest attendance of females via iButton Thermochrons (Maxim, Sunnyvale, CA), which allow distinguishing her times on the nest (i.e. on-bouts) from times of her absence (i.e. off-bouts). Loggers were programmed to take readings at 3 min intervals, with a precision of 0.0625°C, and were placed in the nests once at least three eggs had been laid, and before clutch completion (i.e. before the onset of incubation). Several iButtons were displaced from the actual nest cup, and therefore not all recorded distinct incubation traces, necessitating a data-cleaning step prior to analysis (see electronic supplementary material). After removing these observations (*N* = 335 days), 102 days of incubation were analysed from forest ALAN boxes, 104 from forest control boxes and 89 from urban boxes.

The incR R package [25] was used to determine daily activity onset (inferred from the time of the first incubation off-bout of the day) and activity end (inferred from the time of the last on-bout). We then calculated standardized, relative onset and end of activity relative to sunrise (hereafter ‘onset of activity’) or sunset (hereafter ‘end of activity’), as done in previous studies [7,26]. To quantify restlessness at night, we used the variance of the nest temperature during night-time, defined to last from 22.00 to 03.00 h when all birds were inside their nest-boxes.

### (d) Data analysis

All data analysis was performed in R [27] using the function *lmer* in the package lme4 (v. 1.1–34); [28]. To determine treatment effects on incubation timing, we ran three linear mixed effect models with the following response variables: (1) relative onset of activity (first incubation off-bout time minus sunrise time, in minutes), or (2) relative end of activity (last incubation off-bout time minus sunset time, in minutes) or (3) log-transformed variance in night-time nest temperature. Every model contained nest-box identity as a random intercept. In each model we included nest-box treatment ('urban’, ‘forest control', 'forest ALAN'), clutch size (as a continuous predictor), mean daily ambient temperature (as a continuous linear predictor), date of incubation start (as a linear and quadratic term) and days to hatching (as a linear and quadratic term). Additionally, models included the interactions between (linear and quadratic) date of incubation start and experimental treatment, and (linear and quadratic) days to hatching and experimental treatment. Date of recording was coded as the number of days from the 1 April in each year. When quadratic terms were included, and to improve model convergence, quadratic and associated linear effects were modelled as orthogonal polynomials of degree two using the R function *poly*. The statistical significance of every model predictor was tested via likelihood-ratio tests (LRT). If interactive terms were not significant (including quadratic terms), they were dropped from the model; otherwise, we present results and base statistical inference on full models including all fixed effects. $R_{\mathrm{marginal}}^{2}$ (i.e. per cent of variation in the response term explained by the fixed effects) and $R_{\mathrm{conditional}}^{2}$ (i.e. per cent of variation in the response term explained by the fixed and random effects) were calculated using the R function *r.squaredGLMM* as implemented in the R package MuMIn (v. 1.47.5, [26]). The code to reproduce all analyses is available at: https://zenodo.org/record/8021624 [29].

## 3. Results

**Figure 1.**
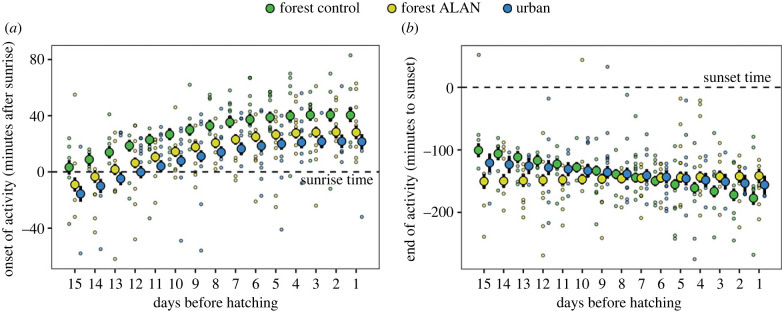
Urban and ALAN effects on relative timing during incubation. Relative time of (*a*) onset and (*b*) offset of activity throughout incubation in female great tits belonging to three treatment groups: control forest females (forest control), forest females exposed to ALAN (forest ALAN), and urban females (urban). Dashed lines illustrate sunrise and sunset times (i.e. *y* = 0). Small points represent raw data, while larger points and whiskers provide mean model predictions ± 1 s.e.

Activity onset over the study period ranged from 04.17 until 06.15 h, and relative to the sun's position, from 62 min before sunrise until 83 min after sunrise (figure 1*a* and electronic supplementary material, figure S1; 295 days of incubation with onset of activity data in 36 nest-boxes). Overall, forest ALAN females and urban females advanced their onset of activity by 14.12 (±4.61 s.e.) and 16.42 (±5.16 s.e.) min, respectively, compared to forest control females (electronic supplementary material, table S3). Throughout the incubation period of sampled nests (i.e. using number of days prior to hatching rather than the absolute start date of incubation), the experimental ALAN treatment of forest females caused an advancement in activity onset which closely mirrored the pattern for urban females (figure 1*a*). The start date of incubation correlated positively with average onset of activity in the forest for both ALAN and control females: the later the incubation start date, the later after sunset females started activity. Conversely, incubation start date correlated negatively with activity onset in urban females, so that late-incubating females started their day earlier ($\chi_{2}^{2}=9.27$, *p* = 0.010; electronic supplementary material, table S3; figure S2). Ambient temperature and clutch size did not impact onset of activity in any group (electronic supplementary material, table S3).

**Figure 2.**
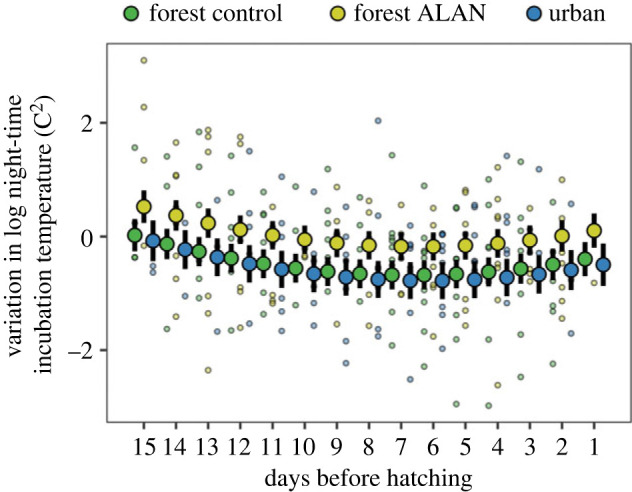
Variation in night-time incubation temperature in the three treatment groups throughout incubation. Night-time incubation temperature variation decreases in the first half of incubation to then level off and the three study groups of females did so in a similar way. However, ALAN-exposed females showed consistently higher night-time incubation temperatures than females in the other two control groups. For details, see figure 1.

The end of activity ranged from 16.49 until 22.02 h, and relative to the sun's position, from 275 min before until 51 min after sunset (figure 1*b*, electronic supplementary material, figure S1, table S4; 279 days of incubation with end-of-activity data in 36 nest-boxes). The activity of forest control females ended earlier as the incubation stage progressed, in contrast to forest ALAN females and urban females whose end of activity remained stable throughout the incubation period ($\chi_{2}^{2}=14.34$, *p* < 0.001; electronic supplementary material, table S4; figure 1*b* and figure S3). Ambient temperature, clutch size and date of incubation start did not impact end of ctivity in any experimental group or habitat (electronic supplementary material, table S4).

Night-time nest temperature variance ranged from 0.05 to 22.20 C^2^ (electronic supplementary material, table S1 and figure S1; 258 nights of incubation with data for variance in incubation temperature in 35 nest-boxes). While all female groups showed a U-shaped pattern of variation in night-time incubation temperature in relation to absolute date, variation was consistently higher in forest ALAN females than in the other groups ($\chi_{2}^{2}=6.33$ , *p* = 0.042; electronic supplementary material, table S5; figure 2). Ambient temperature and clutch size did not impact night-time nest temperature variance in any experimental group or habitat (electronic supplementary material, table S5).

## 4. Discussion

Our experimental manipulation allowed us to show that the previously reported [16,21] difference in timing of behaviour between urban and forest bird populations can be largely explained by light pollution rather than by other co-varying urban factors. ALAN effects as observed in the forest explained part of the advanced morning activity of urban females, which were exposed to environmental ALAN outside of their nests. In accordance with previous findings, our results support experimental evidence obtained from captive studies, which suggested that exposure to ALAN advances the morning onset of activity [8,30]. We also found that onset of activity became later relative to sunrise as hatching date approached, in accordance with the previously reported seasonal decline in ‘earliness’ of songbirds during the reproductive period [31]. The different responses of activity onset of urban and forest birds to later laying date are currently unexplained, but might relate to urban food constraints or other features of city life that cause females to rise progressively earlier [23].

The end of activity was also significantly affected by the ALAN treatment. Forest females exposed to ALAN behaved more similarly to urban females than forest control females, as neither forest ALAN nor urban females modified the end of activity over the incubation period. Conversely, in forest control females the end of activity became earlier over time. Combined with the results on onset of activity, the duration of night rest increased as hatching date approached in forest females. This pattern was, however, weaker in both ALAN-exposed forest females and urban females. Thus, our results clearly show that urbanization and ALAN disrupt natural patterns of incubation behaviour in great tits.

We also found that the ALAN treatment consistently increased variation in night-time temperature in the forest environment. This suggests that ALAN can disrupt the natural nocturnal rest during incubation, a result that is in line with previous experimental findings for great tits during territory establishment [10], as well as during chick provisioning [11]. However, the increased variation in ALAN forest birds did not match the behaviour of urban females, whose levels of night-time temperature variation instead were similar to forest controls. One reason could be that forest ALAN birds were exposed to constant, albeit low intensity, 24 h light inside their nest-box, which might have affected restlessness and sleep patterns, whereas urban birds were largely sheltered from ALAN in their nest-boxes. Moreover, forest ALAN birds, who had little previous exposure to light pollution, might have needed time for physiological and behavioural adjustments to the sudden exposure to ALAN. Conversely, urban females had been exposed to urban environmental conditions since hatching, and the urban population as a whole might be accustomed to such conditions through either habituation (phenotypic plasticity) or adaptation (genetic change) [32].

Since changes to incubation behaviour may alter the post-hatching development of offspring, for instance in terms of growth rate [15,22], energy metabolism [15], cold tolerance [33] and reproductive success [27], there may be future costs associated with the change in incubation rhythms observed in this study. An experimental study on starling hatchlings (*Sturnus vulgaris*) reported that rhythmic low-intensity light (10 lx) synchronized perinatal melatonin levels [34]. While it is not clear how much ALAN enters nest-boxes, a similar experimental study in wild great tits has shown that even low levels of ALAN can alter melatonin cycles [35]. Furthermore, incubating females may also suffer consequences of ALAN during this costly reproductive stage. For example, an earlier onset of activity could mean that timing is not optimally synchronized to foraging opportunities, or that birds are more at risk of predation [36]. However, parallel evidence from our system suggests that early onset of activity in great tit females is associated with an increase in the number of nestlings produced [21]; whether this also corresponds to higher recruitment rate is currently unknown.

Overall, our results suggest that ALAN can explain most of the difference in onset of activity observed between forest and urban great tit females. However, since nest-boxes may shield nestlings and adults from light pollution [37], it is possible that ALAN effects in the city affect incubation indirectly. Possibly, ALAN exposure of urban birds outside nest-boxes could trigger community-level changes in activity that affect females inside the boxes, for instance through shifts in dawn chorus. Nevertheless, if ALAN effects can be seen in cavity-nesting species, these are likely to be even stronger in open-cup nesting species that have rarely been studied in the context of urbanization and light pollution [37,38]. Overall, individual fitness consequences of ALAN and potential cascading population effects are largely understudied and need to be addressed as, natural darkness is under threat globally.

## Data Availability

The dataset and R scripts needed to reproduce the analyses presented in the paper are available from the Zenodo repository: https://zenodo.org/record/8021624 [29]. Additional information are provided in the electronic supplementary material [39].
